# Demographics and complaints of university students who sought help at a campus mental health service between 1987 and 2004

**DOI:** 10.1590/S1516-31802008000100011

**Published:** 2008-01-03

**Authors:** Maria Lilian Coelho de Oliveira, Clarissa de Rosalmeida Dantas, Renata Cruz Soares de Azevedo, Cláudio Eduardo Muller Banzato

**Keywords:** Mental health, Counseling, University health services, Mental health services, Universities, Saúde mental, Aconselhamento, Serviços de saúde para estudantes, Serviços de saúde mental, Universidades

## Abstract

**CONTEXT AND OBJECTIVE:**

Client characterization is an important step in evaluating the services offered by campus counseling and mental health centers and in their further planning and development. The objectives here were to describe reported complaints and demographics among students who sought counseling/mental healthcare at a Brazilian campus mental health service over a 17-year period and to compare these characteristics with those of the general university student body.

**DESIGN AND SETTING:**

Retrospective study at the Psychological and Psychiatric Service for Students (SAPPE), Universidade Estadual de Campinas (Unicamp).

**METHODS:**

The participants were all of the 2,194 students who sought counseling/mental health care at SAPPE from 1987 to 2004. Information was obtained from clients’ clinical charts. Unicamp's database was consulted for general information on its students.

**RESULTS:**

The findings indicated overrepresentation, among the clients, of undergraduates, female students, students from Brazilian states other than São Paulo, students living in the campus residence hall and those whose main source of income was a scholarship grant. We also found overrepresentation of Humanities and Arts students among the clients. The most frequently reported complaints were difficulties in interpersonal relationships, family conflicts and poor academic performance.

**CONCLUSION:**

Course level (undergraduate or postgraduate), study field, living in a university residential facility and reliance on a scholarship grant were found to influence the behavior of seeking mental health counseling among Brazilian university students in this study. Course level was found to influence the pattern of complaints reported at first contact with the mental health service.

## RESUMO

**CONTEXTO E OBJETIVO:**

A caracterização dos pacientes é uma etapa fundamental para a avaliação e aprimoramento dos serviços de saúde mental para estudantes universitários. Os objetivos deste estudo foram descrever as características sócio-demográficas e as queixas referidas pelos estudantes de uma universidade pública brasileira que procuraram atendimento num serviço de saúde mental para universitários por um período de 17 anos e compará-los com a totalidade dos estudantes dessa universidade.

**TIPO DE ESTUDO E LOCAL:**

Estudo retrospectivo realizado no Serviço de Assistência Psicológica e Psiquiátrica ao Estudante (SAPPE) da Universidade Estadual de Campinas (Unicamp).

**MÉTODO:**

Os sujeitos foram 2.194 estudantes que procuraram atendimento em saúde mental no SAPPE no período de 1987 a 2004. Os dados foram obtidos a partir de seus prontuários. O banco de dados da Unicamp foi consultado para a obtenção das informações sobre os estudantes da universidade como um todo.

**RESULTADOS:**

Entre os alunos que procuraram atendimento encontramos uma sobre-representação de estudantes de graduação, do sexo feminino, procedentes de outros estados brasileiros que não o Estado de São Paulo, que moravam na moradia universitária, que tinham bolsas de auxílio como principal fonte de renda e das áreas de Humanas e Artes. As queixas mais relatadas foram: dificuldades no relacionamento interpessoal, conflitos familiares e desempenho acadêmico insatisfatório.

**CONCLUSÃO:**

O nível do curso (graduação ou pós-graduação), a área do curso, ser residente na moradia universitária e possuir bolsa de auxílio foram variáveis que influenciaram a procura de atendimento em saúde mental dos estudantes universitários brasileiros avaliados. O nível do curso influenciou o tipo de queixa referida no primeiro contato com o serviço de saúde mental.

## INTRODUCTION

As early as in the 1920s, the mental health of university students began to be a matter of concern in developed countries,^1^ which eventually led to increased awareness of the fact that post-secondary school students face not only the challenges of higher education, but also many developmental issues that accompany late adolescence and young adulthood. Among these are their individuation and connectedness to their families, the development of friendships and intimate relationships, career choices and the pursuit of personal and professional goals. In addition, it should be noted that many young adults experience their first psychiatric episode during their time at university, considering that epidemiological studies indicate that many mental illnesses usually have their onset in young adulthood.^2^ More recently, the mental health of postgraduate students has also gained attention and studies have been reporting high prevalence of depression, distress and burnout among postgraduates.^3-5^

In the 1950s, many campus counseling/mental health services were founded in universities in the United States and Europe.^1^ Likewise, the first mental health service devoted to university students in Brazil was established in 1957, in the Medical School of the University of Recife.^1^ However, it was only in the late 1980s that campus counseling/mental health services spread among the large universities of the State of São Paulo.^6^ Several of these services focus specifically on students in medical and health-related courses.

Universidade Estadual de Campinas (Unicamp), a public university in the State of São Paulo, Brazil, established a campus mental health service in 1987: the Psychological and Psychiatric Service for Students (Serviço de Assistência Psicológica e Psiquiátrica ao Estudante, SAPPE). This is designed to provide clinical care for regularly enrolled students from all university courses, both undergraduates and postgraduates (i.e. students on master's and doctoral programs).

The following are some of the main characteristics of SAPPE: a) service mission clearly stated as the provision of broad-based mental health clinical care (i.e. not limited to academic issues); b) an open-door policy, such that students are typically self-referred or referred by fellow students who had a previous contact with the service; c) confidentiality as a central concern, such that students are assured that the information about their treatment is confidential and that any kind of report would be made available only on their own request and in their best interest; and d) a two-pronged therapeutic approach: one focused on brief interventions – such as brief psychotherapy (up to 24 sessions), very brief psychotherapy (4 sessions), and one-session emergency attendance – which are believed to be suitable for most of SAPPE's clients;^7^ and another focused on continuity of care, in order to assure effective treatment and support for clients with severe mental health problems.

In response to the perceived increase in the frequency and complexity of students’ demands for mental healthcare, Unicamp has allocated more resources towards the development of SAPPE over the years, and consequently the service's material and human resources have improved. Accordingly, the number of sessions performed at SAPPE increased from 2,700 in 1995 (the first year for which this information was available) to 9,616 in 2003, including both undergraduate and postgraduate students.

Client characterization is an important step in evaluating the services offered by campus counseling and mental heath centers and in their further planning and development.^8^

## OBJECTIVE

The objectives of the present study were to describe the demographic characteristics of students who sought counseling/mental healthcare at a campus mental health service over a 17-year period, to compare these characteristics with those of the general student body of the university and to describe undergraduate and postgraduate students’ complaints at their first contact with the service.

## METHODS

The present retrospective study was approved by the Research Ethics Committee of Faculdade de Ciências Médicas, Universidade Estadual de Campinas (FCM-Unicamp). The participants were 2,914 undergraduate and postgraduate students who sought counseling/mental health care at the campus mental health service (SAPPE) of Unicamp. All student-clients seen for at least one session between March 1987 and March 2004 were included in the study.

Data were obtained from the clients’ clinical charts, and specifically from the records of the semistructured interview that was conducted by staff members when the student sought help at SAPPE for the first time. The instrument applied during these interviews was developed by the campus mental health service staff in order to record demographic characteristics, information about the client's family, the client's complaints and concerns and the therapeutic plan. Over the 17 years covered by this study, the interviews were conducted by two senior staff members (both clinical psychologists; one of them is the first author of the present study) who were in charge of attending the clients at the time of their first contact with the service.

The general demographic characteristics of all undergraduate and postgraduate (master's and doctoral) students regularly enrolled in the university from 1996 (when such data were first available) to 2004 were obtained from Unicamp's database.^9^

The data collected were entered into a specific database using the Statistical Package for the Social Sciences (SPSS 9.0). To analyze differences between undergraduates and postgraduates we used the Mann-Whitney test for continuous variables and the chi-squared test for nominal variables.

## RESULTS

The mean age of the student-clients was 23.3 years (standard deviation, SD = 5.1; median = 22 years); 40.4% were male (n = 1178) and 59.6% (n = 1735) were female. Undergraduates made up 75.6% of student-clients and postgraduates accounted for 24.4%.

Campinas, the city where both the main university campus and the mental health service are located, was the home of 21.1% of SAPPE's clients, while 52.6% of them came from other cities within the State of São Paulo, 24.4% came from other Brazilian states and 1.9% came from other countries (1.1% of the undergraduate clients and 4.5% of the postgraduate clients). The housing arrangements of SAPPE's clients were that 35.0% were living with roommates in a rented apartment or house; 32.2% were living with their families (parents, relatives or husband/wife); 18.5% were living in the campus residence hall; and 14.3% were living on their own.

Some of the demographic characteristics of the student-clients are presented and compared with the general characteristics of Unicamp students (assessed during their first year in the University, from 1996 to 2004) in Table 1. This shows that undergraduate students were overrepresented among SAPPE's clients, and especially female undergraduates. Students who came from Brazilian states other than São Paulo, those who were living in the campus residence hall and those whose main source of income was a scholarship were also overrepresented among the campus mental health service clients. Regarding the study field, we found an overrepresentation of Humanities and Arts students. Exact Sciences, Technology and Engineering undergraduate students were slightly underrepresented, but postgraduate students from those study fields were not. Biological and Medical Sciences students in general were underrepresented among SAPPE's clients.

**Table 1.** Characteristics of campus mental health service clients compared with the general characteristics of students at Universidade Estadual de Campinas (Unicamp)

	SAPPE's clients (n = 2914)		All students at Unicamp (n = 46160)	
	Undergraduates 75.6 % (n = 2203)	Postgraduates 24.4% (n = 711)	Undergraduates 51.1 % (n = 23579)	Postgraduates 48.9% (n = 22581)
**Mean age in years (median)**	21.7 (21)*	28.4 (27)*	NA	NA
**Female**	58.5%†	62.9%†	33.2%	NA
**Married**	5.2%‡	25.0%‡	4.5%	NA
**Students from Brazilian states other than São Paulo**	19.9%	46.4%	7.5%	30.7%
**Living in the campus residence hall**	21.6%	8.6%	5.5%	1.4%
**Scholarship grant as the main source of income**	43.0%‡	81.7%‡	11.4%	41.8%
**Mean monthly income (in Brazilian minimum monthly salaries)**	2.5*	6.5*	NA	NA
**Study field**				
Exact Sciences, Technology and Engineering	45.2%	48.4%	56.2%	47.9%
Biological and Medical Sciences	13.8%	14.7%	19.1%	22.8%
Humanities	29.5%	33.1%	18.9%	26.6%
Arts	11.5%	3.8%	5.8%	2.7%

SAPPE = Psychological and Psychiatric Service for Students (Serviço de Assistência Psicológica e Psiquiátrica ao Estudante); NA = data unavailable in Unicamp's database.

* Mann-Whitney test, p < 0.0001;

† chi-squared test, p = 0.0395;

‡ chi-squared test, p < 0.0001.

The most frequently reported complaints that led the students to seek help were difficulties in interpersonal relationships, family conflicts and poor academic performance. Table 2 presents all the complaints reported by at least 10% of SAPPE's clients. Although conflicts within couples were reported by only 5.7% of all of SAPPE's clients, they were the second most frequently reported complaint among postgraduate clients (reported by 23.3% of postgraduates).

**Table 2.** Most frequently reported complaints of students at Universidade Estadual de Campinas (Unicamp)

Complaint	All of SAPPE's clients* (n = 2914)	Undergraduates† (n = 2203)	Postgraduates‡ (n = 711)
**Difficulties in interpersonal relationships**	31.4%	31.2%	32.1%
**Family conflicts**	23.8%	25.0%	19.8%
**Worry about professional future**	21.1%	21.5%	19.8%
**Poor academic performance**	18.9%	19.8%	16.4%
**Feeling down**	16.6%	16.2%	17.9%
**Difficulty in making friends**	14.5%	15.0%	13.0%
**Lack of self-confidence**	14.0%	13.6%	15.1%
**Difficulties in talking about oneself**	12.9%	12.9%	12.9%
**Lack of motivation**	12.5%	12.8%	11.6%
**Doubts about course choice**	11.8%	14.3%	4.1%

* Percentage of all of clients of the Psychological and Psychiatric Service for Students (Serviço de Assistência Psicológica e Psiquiátrica ao Estudante, SAPPE) who reported the complaint;

† Percentage of undergraduate clients of SAPPE who reported the complaint;

‡ Percentage of postgraduate clients of SAPPE who reported the complaint.

Only 2.4% of the student-clients (2.7% of undergraduates and 1.6% of postgraduates) reported problems relating to recreational drug use at their first contact with the service. Severe conditions that could suggest psychotic illness were reported by 1.0% of the student-clients. Suicidal thoughts were the expressed complaint of 3.0% of the student-clients (3.2% of undergraduates and 2.5% of postgraduates) and 28 students (25 undergraduates and 3 postgraduates; 1.0% of the clients) sought help at the campus mental health service right after a suicide attempt. Sexual abuse was the problem presented by 27 students (16 undergraduates and 11 postgraduates; 0.9% of clients).

## DISCUSSION

There are limitations to the present study, mainly due to its reliance on self-reported complaints recorded on clinical charts for clinical purposes (i.e. no standardized psychopathological assessment was performed) at the very first contact with the service and the lack of information about some general characteristics of the university students. Nevertheless, the data obtained form a valuable report comprising a large number of Brazilian university students within various study fields who were seen in a university counseling and mental health service over a considerable time span.

Regarding student-client demographics, we found an overrepresentation of undergraduate students among SAPPE's clients. One possible explanation for this finding might be that postgraduate students have a higher personal income and thus better access to other mental healthcare providers (through private care or health insurance), thus seeking help at SAPPE less frequently. It is also possible that outreach strategies focused on undergraduate students have affected help-seeking behavior among that particular group.

Information about the campus mental health service is available in the booklet provided to secondary school students when they apply for enrollment on any undergraduate course in the university and also in the students’ guide provided to undergraduate students at the time of enrollment. Considering that the first year at the university may be especially challenging, SAPPE has been participating in the university's welcome program for freshmen.

There is no special outreach strategy targeting postgraduate students. Moreover, postgraduate students tend to be in closer contact with their thesis advisors and research groups and less integrated with the general campus community. They are therefore perhaps less familiar with the services offered by the university. Another possible explanation why postgraduate students were underrepresented among SAPPE's clients might be that psychological mental health problems are less common among that student group. Any of the hypotheses mentioned above would require further investigation.

Women were overrepresented among undergraduate student-clients, in relation to the general university undergraduate student body. General data on the gender of Unicamp's postgraduate students was unavailable, but we suspected that females were overrepresented among postgraduate clients too. Gender differences in help-seeking behavior, such that women are more open to seeking professional help, are well known and they have been consistently replicated across demographic groups and national boundaries.^10,11^

Students with certain demographic characteristics, such as living in the campus residence hall and relying mainly on a scholarship grant for funding, were also overrepresented among campus mental health service clients. It is important to note that availability of campus residence facilities at Unicamp is limited and students undergo selection based on social and financial criteria, in order to be allowed to live there. The same criteria are adopted for distributing a proportion (48.1%)^9^ of the scholarships grants provided to undergraduate students by Unicamp. Our findings suggest that the role of SAPPE as a mental healthcare provider might be even more important to students with a worse economic situation (undergraduates and postgraduates who are eligible for living in the campus residence and undergraduates eligible for scholarship grant), since they may have limited access to private care or health insurance. These results differ somewhat from another study that evaluated 2785 undergraduate and postgraduate students and found that lack of perceived need, skepticism about treatment effectiveness and low socioeconomic background were predictors for not receiving mental healthcare.^12^

Scholarship grants provided to postgraduate students are offered mainly by governmental funding agencies and their distribution follows academic/scientific criteria. On the one hand, such grants might provide some financial security during the postgraduate course. On the other hand, however, they might represent an additional pressure on students, since funding agencies impose exclusive dedication to research, restricted time frames and higher academic performance requirements. It has been reported that this scenario is associated with feelings of insecurity, anxiety, distress and even burnout.^4^ Furthermore, it might be associated with our finding of an overrepresentation of postgraduate students whose source of income was a scholarship grant among SAPPE's clients.

We also found among our clients that there was an overrepresentation of students from Brazilian states other than the one where the University is located. Students who live far from their families possibly rely more on SAPPE as part of their support network.

Regarding the complaints profile, we found that the most frequent complaints were similar to those presented by university students in other countries.^8,13,14^ In other studies, the complaints mainly comprise problems in the field of interpersonal relationships and worries about academic and career-related issues.

Wagner et al.^15^ found that the prevalence of any illegal drug use over the last 12 months was 34.2% for male students and 27.1% for female students at the Universidade de São Paulo (USP), Brazil, in 2001. The prevalence of any illegal drug use over the last 30 days was 24.9% for males and 17.5% for female students. Similar results were found in a broad survey carried out recently at Unicamp (Marly Coelho Carvalho Neves, personal communication). Unsurprisingly, the frequency of drug use-related problems reported at the students’ initial contact with SAPPE was much lower than the figures from the epidemiological field studies mentioned earlier. There are several likely reasons for this difference. Firstly, denial of the adverse consequences of psychoactive drugs at some level is itself part of recreational drug use behavior. In addition, the frequency of self-reported drug use-related problems might be affected by the observed trend towards an increased consumption and endorsement of several substances among Brazilian undergraduate students.^16^ Even among those who acknowledge a drug-related problem, it may the case that they avoid seeking help at a campus facility due to confidentiality and legal concerns.

The frequency of severe mental heath conditions found in our study is in accordance with reports from other countries on students’ mental health.^13,14,17^ Although they may be few in number, students with serious mental health illness or psychological problems are highly demanding in terms of staff time and resources and they are especially challenging to campus mental health services.^2,15^ A recent study suggested that campus mental health services can possibly reduce the delay between the onset of psychiatric conditions and its proper treatment. In addition, it should be noticed that mental health status in young adulthood is associated with substance use, academic problems and other detrimental social outcomes later in life.^12^ Thus, we think that campus mental health services must be a policy priority.

## CONCLUSION

Course level (undergraduate or postgraduate), study field, living in a university residential facility and reliance on a scholarship grant were found to influence the behavior of seeking mental health counseling among the Brazilian university students in this study. Course level was found to influence the pattern of complaints reported at the first contact with the mental health service.
